# Housing ableism in finding and maintaining housing among people with disabilities: A scoping review

**DOI:** 10.1371/journal.pone.0351309

**Published:** 2026-06-10

**Authors:** Sally Lindsay, Gözde Öncil, Sarah Leo

**Affiliations:** 1 Bloorview Research Institute, Holland Bloorview Kids Rehabilitation Hospital, Toronto, Ontario, Canada; 2 Department of Occupational Science & Occupational Therapy, University of Toronto, Toronto, Ontario, Canada; 3 Rehabilitation Sciences Institute, University of Toronto, Toronto, Ontario, Canada; The Chinese University of Hong Kong, Shenzhen, CHINA

## Abstract

**Background:**

Access to safe and affordable housing is a human right; however, people with disabilities are more likely to live in inaccessible, insecure, unaffordable and poor-quality housing than people without disabilities. They often experience many forms of disability-related discrimination (ableism) in finding and maintaining adequate housing. The objective of our study was to synthesize the literature on housing ableism among people with disabilities seeking or maintaining independent housing.

**Methods:**

We conducted a scoping review that involved searching six international databases that identified 10,082 studies, 52 of which met our inclusion criteria involving empirical research, without language or date restrictions, that had a sample focusing on people with disabilities, that had findings related to independent housing and ableism. We followed the best practices of scoping review methodology and applied an inductive data analysis approach.

**Results:**

The studies included in the review involved 13 countries over a 47-year period. Our review identified the following findings: (1) ableism in finding and maintaining appropriate housing through renting and ownership (i.e., lack of suitable housing, barriers to accessing or viewing properties, affordability); (2) factors affecting housing ableism (i.e., lack of knowledge about the needs of people with disabilities, and intersectional factors (i.e., socio-economic status, type of disability, housing type, systemic and policy barriers); and (3) the impact of housing ableism (i.e., challenges with neighborhood environment).

**Conclusions:**

Our findings highlight the extent to which people with disabilities encounter multiple forms of discrimination in trying to find suitable housing. Our review emphasizes the urgent need to reduce the ableism that people with disabilities by addressing structural barriers and prioritizing housing accessibility. Policy reforms, increased attention and investment towards accessible housing are needed for a more inclusive housing sector.

## Introduction

The global housing affordability crisis limits people’s ability to suitable housing due to high costs, housing shortages, and limited accessible options [1–3]. The United Nations Flagship Report on Disability and Sustainable Development Goals identifies housing for people with disabilities as a major challenge over the next decade [4]. People with disabilities represent approximately 16% of the global population and one of the world’s largest minoritized groups worldwide [5]. The United Nations Convention on the Rights of Persons with Disabilities [6] recognizes access to safe, secure, adequate, accessible and affordable housing as a fundamental human right and a key social determinant of health. Despite this, people with disabilities often face limited housing options, and are more likely to rent and experience higher rates of homelessness [7–10]. Many live in unaffordable, insecure, or poor-quality housing, including units requiring major repairs, or overcrowded conditions and spend a greater proportion of their income on housing than people without disabilities [9,11–14].

### Housing discrimination among people with disabilities

People with disabilities often experience stigma or discrimination when seeking or maintaining housing [15]. Disability-related discriminatory attitudes, behaviours and practices, collectively referred to as ableism, create barriers to equitable, high-quality housing [16]. Ableism includes actions or conditions that devalue and disadvantage people at individual, organizational and structural levels [17]. Housing ableism operates across these levels by limiting access to safe and affordable housing through structural, spatial, economic and attitudinal barriers [18,19]. Direct discrimination includes discriminatory comments or behaviours, whereas indirect discrimination includes withholding information, imposing unclear policies, or restricting access to resources [1,20]. Negative assumptions about disability can reinforce discrimination in the housing sector [21]. Housing providers, including landlords, realtors and builders, often lack knowledge about accessibility needs and may impose barriers during the housing search process [1,20,22]. For example, people with disabilities often encounter challenges when providers fail to share information, complicate appointments, deny modification requests, or create barriers in navigating rental processes [20,23]. Builders may also overlook universal design principles or resist incorporating accessibility features due to perceived costs or effort [1,22]. These practices contribute to a shortage of suitable housing and reinforce structural forces of ableism [10]. As a result, people with disabilities often experience unsafe or substandard living conditions, including risks related to neighbourhood safety, sanitation, and environmental hazards [18,24,25].

### Knowledge gaps and significance

Housing systems face increasing pressure due to rising development costs, interest rates and a limited supply of accessible and affordable housing [10,26]. These pressures may intensify discrimination against people with disabilities [16]. Existing research primarily focuses on populations in institutional or supported living settings, such as individuals with severe mental health conditions, older adults, or those living in a group homes [10,27–34]. However, many people with disabilities prefer independent housing because it supports autonomy, self-determination and social inclusion [35]. Independent housing refers to limitations outside institutional settings, including private family homes. Access to adequate housing can improve physical and mental health, while inadequate housing can increase risk of injury, isolation and stress [2,36,37]. Poor housing conditions also limit access to services and reduce participation in education and employment [10,22,27,38,39]. Despite its importance, no reviews have synthesized evidence on housing ableism experienced by people with disabilities seeking or maintaining independent housing. This review addresses this gap by examining the extent, nature and impacts of housing ableism across diverse contexts.

## Materials and methods

We conducted a scoping review to examine the international literature on housing ableism experienced by people with disabilities seeking or maintaining independent housing. We followed established scoping review guidelines [40–42] and adhered to the Preferred Reporting Items for Systematic Reviews and Meta-Analysis, extension for Scoping Reviews (PRISMA-ScR) (see S2 Fig.) [43]. A scoping review is appropriate given the breadth, heterogeneity and interdisciplinary nature of the literature, allowing us to map key concepts, evidence sources and research gaps without restricting inclusion by design, location, or time period [40].

Our synthesis is conceptually informed by the social model of disability and a social ecological perspective, which together situate housing ableism as arising from interactions between structural, environmental, and individual factors [44,45]. The social model of disability conceptualizes disability as arising not from individual impairments, but from societal, structural and environmental barriers that restrict participation and inclusion [44]. In contrast, the socio-ecological model emphasizes that these barriers operate across multiple interacting levels, including individual, interpersonal, community and policy, influencing experiences and outcomes in complex and interconnected ways [45]. Consistent with scoping review methodology, we applied an inductive analytical approach rather than a predefined theoretical framework [40–42].

### Search strategy and data sources

We developed the search strategy, in consultation with a research librarian and experts in disability, ableism and housing. The following databases were searched: Ovid Medline, Healthstar, Embase, PsychInfo, Scopus, and Web of Science. The search involved the following key elements: *Population* referring to people with disabilities (based on the definition in the World Health Organization’s framework of International Classification of Functioning, Disability and Health, “an umbrella term for impairments, activity limitations and participation restrictions” [46]); *Concept* referring to ableism (discrimination towards people with disabilities that occurs directly or indirectly at individual, organizational or structural levels, which can include attitudes, behaviours, policies, and practices (e.g., barriers to accessing housing, refusing to allow for home modifications [47])); *Context* independent housing including private residences or living with family outside of institutional settings) (see S1 Fig. for search strategy). We also reviewed the reference lists of all included articles to identify potential additional studies.

### Article selection

The inclusion criteria involved the following: (1) focused on people with disabilities; (2) used empirical methods (either qualitative or quantitative) that reported at least one finding related to housing (i.e., independent housing or living with a family member) and ableism; and (3) peer-reviewed publication published up to May 2025 without language restrictions. One article in our review was translated into English using *Google* translate, which was verified for accuracy by a team member who is fluent in the language. Exclusion criteria involved: (1) non-peer reviewed and grey literature (books, book reviews, conferences, editorials, theses) because they are at risk of bias; and (2) studies focusing on nursing homes, residential care or group homes, because our focus is on independent housing [28,29,31–33].

**Fig 1:**
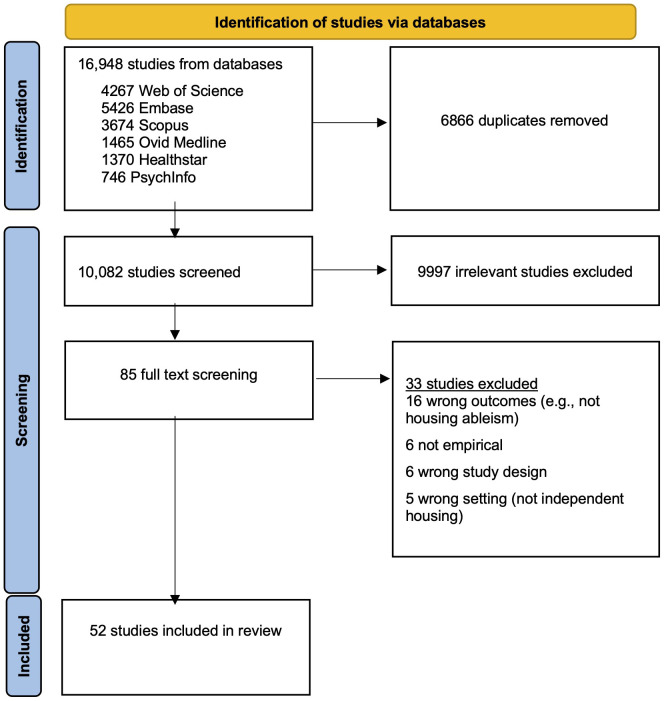
Overview of the search process.

The first author conducted the search and imported the articles into *Covidence* for screening and data extraction. After removing duplicates, two authors independently screened 10,082 titles and abstracts while applying the inclusion criteria, of which 9997 were deemed irrelevant. Next, two authors screened 85 full-text articles that met our inclusion applying the criteria independently to ensure consistency. After screening, 52 studies were identified as meeting all our criteria for the final review (see Fig 1 for search process and reasons for exclusion). When disagreements arose about whether articles met the inclusion criteria, the authors discussed them until reaching consensus.

### Data extraction and synthesis

We used an inductive approach to data extraction and synthesis, consistent with scoping review methodology [40–42]. We developed and piloted our data extraction form to ensure consistency. Two researchers independently extracted data, and a third author verified accuracy. We discussed any discrepancies until consensus was reached. We applied an open coding approach [42] to identify patterns related to housing ableism (e.g., rates, types of ableism). We grouped findings into thematic categories and synthesized results across studies using descriptive summaries and qualitative content analysis. We also reported trends by participant characteristics, disability type, and housing context, where possible.

## Results

### Study characteristics

We identified 52 studies spanning 13 countries over a 47-year period (see Table 1). Most studies were conducted in high-income countries, particularly the United States, United Kingdom and Australia. Thirty-eight percent focused on specific types of disabilities, 42% included mixed disability types and the remainder did not specify disability type. Participant characteristics varied widely. Studies included adults [48–68], children [51,69–71], households [24,72–76] and service providers, although many did not report key demographics. Among studies reporting gender most included predominantly female samples [48,49,51,53,54,56,57,61,65,69,77,78], and none reported including non-binary participants.

**Table 1:** Overview of study characteristics.

Author, year (Country)	Sample characteristics** (age, sex, disability type, race, housing type)	Design and analysis (measures and theory)	Key findings*
Anderson et al. 2024 (US) [79]	**Socio-demographics and sample size not provided	Secondary analysis of 2020 American Community Survey (housing issues, housing type) -No theory used	-Adults with disabilities are significantly more likely to live in old housing compared to those without a disability, and issues with poor housing -Adults with disabilities are significantly more likely to live in apartments or mobile homes than adults without disabilities
Aplin et al. 2015 (Australia) [48]	**55 participants: 42 households, 13 joint interviews −36 people with disabilities −5 parents/ guardians −13 spouses −1 carer Mean age: 64, age range: 25–87, 55% female	Interviews (home modifications, decision making, social meaning of home environment) -No theory used	-Home modifications are mostly positive for clients and families, depending on involvement in decision- making, workmanship of the builders and societal dimension -Heightened sense of permanency but negative physical dimension of home (hard to heat up because of modifications etc.)
Aplin et al. 2020 (Australia) [49]	**13 adults with disabilities (physical, sensory, cognitive, psychological), 2 spouses (6 males, 9 female; mean age 54.4), age range: 39–64	Interviews (meaning of home) -Ontological security theory	-People with disabilities describe a loss of control over location, condition, modification and maintenance of their home
Aranda 2015 (US) [23]	**1665 housing providers contacted by paired testers -Two tests: people who are deaf or hard of hearing and control case -People who use a wheelchair and control case	Pair-test methodology via telephone or the internet (discrimination, housing modifications, disability type) -No theory reported	-People who are deaf or hard of hearing face barriers during the home seeking process -People who use wheelchairs face barriers at several points including finding accessible units, securing appointments, being shown units, and receiving a response to reasonable modification requests
Beer et al. 2020 (Australia) [80]	**Socio-demographics and sample size not provided	Secondary analysis of Household, Income and Labor Dynamics in Australia Survey data (disability, index of relative homelessness risk; -No theory reported	-Homelessness risk is not reduced for people with disabilities after the implementation of National Disability Insurance Scheme -Homelessness risk is significantly higher for disabled people compared to general population -Overrepresentation of people with cognitive disabilities and mental health issues with high relative homelessness risk
Bhakta and Pickerill 2016 (UK) [72]	**5 households and 44 visitors to the community	Mixed methods: site visits, videos, spatial mapping, observations, surveys, interviews (accessibility eco-communities) -Theory: embodiment	-Barriers to implementing accessible policies, lack of knowledge of accessibility regulations, poor attitudes towards access and complacency from builders, lack of knowledge, financial issues regarding costs of implementing accessible features -Changes in accessibility standards affected the building process -Eco-housing design was often not accessible
Burns 2004 (UK) [81]	**31 people with a physical disability Housing type: Homeowners or trying to purchase	Interviews (experiences of buying a house and aspects of home ownership) -No theory reported	-Reactions to being different from house builders -Difficulty accessing properties -House builders lack of knowledge about disability -Builders often view accessibility issues as extra options
Callaway et al. 2021 (Australia) [82]	504 housing property advertisements Housing type: 21% house, 17.7% apartment, 12.7% unit, duplex/villa or townhouse, 46% group homes, 1.6% larger dwelling, 1% other	Audit of housing advertisements (housing characteristics, specialist disability registration and funding) -No theory reported	−45.3% of housing advertisements were not accessible to people not approved for funding -Lack of clarity regarding the relationship of advertised vacancies to the specialist disability accommodation framework, as less than 1 in 5 vacancies mentioned certificates or design standards
Chakraborty et al. 2023 (US) [73]	**790 households in metropolitan areas (45% had a disability)	Survey (adverse impacts of weather event, subsidized housing) -No theory reported	-Households with people with disabilities were more severely impacted by winter storms than households without people with disabilities
Challe et al. 2023 (France) [50]	Inquiries to 1000 rental apartment property advertisements, majority 1-bedroom (landlords and realtors) Applying with 3 fictitious profiles: French male with no disability; French with visual disability; North African descent name with no disability	Property email enquiries and survey -No theory reported	-Having a visual impairment penalized people by reducing their chances of visiting an apartment -Landlord often requests additional information before offering a visit
Dawkins and Miller 2017 (US) [83]	**Socio-demographics and sample size not provided	Secondary analysis of US department of housing (socio-demographics of people with disabilities living in assisted housing) -Theory: person-environment framework	-Many people with disabilities are served by the housing and urban development department sponsored programs that are not designed for people with a disability −70% of extremely low-income households that include a person with a disability do not receive housing assistance -About 92% of public housing households that include people with a disability do not live in designated units −53% of extremely low-income renter households that include a person with a disability did not receive housing assistance
Dunn 1990 (Canada) [51]	300 people with various disabilities −44% male; 11% under 18, 46% between 18–64, 43% 65+ -Housing type: 29% homeowners, 29% tenants in private housing, 42% tenants in subsidized housing	-Survey (productivity outcomes, living arrangement outcomes) -Theory: independent living paradigm	-Long delays on the wait list for housing modifications −50.6% of tenants with disabilities were concerned about management’s treatment of them −49.2% of tenants with disabilities felt their tenant’s association was responsive to their needs −30% of respondents needed help with getting connected with community services
Flage and Le Gallo 2025 (France) [52]	1750 emails regarding rental housing market, 100% 1-bedroom apartments (4 fictitious profiles of a father without a disability searching for an apartment for his son: person with visual disabilities, accompanied by a guide dog; a person with mental disabilities; a person with motor impairments; a person without disability); 100% male	Email matched-pair procedure correspondence test (invitation to visit/further contact, private vs real estate agent) -No theory reported	-Discrimination against people with visual disabilities accompanied by a guide dog, people with mental health disabilities, and motor impairments in the process of renting a house -Discrimination against applicants increases with the level of rent-discrimination against people with visual disabilities with a guide dog is non-significant for real estate agents, but significant for private landlords due to the existence of a guide dog -Discrimination against people with mental or motor disabilities was significant from both real estate agents and private landlords
Friedman et al. 2018 (US) [24]	**620,000 housing units	Secondary analysis of 2009 American Housing Survey data (neighborhood conditions, housing adequacy) -Theory: spatial assimilation model and discrimination model	-Residential disadvantage among households with people with disabilities is worse in the sales market compared to the rental market -Households with people with disabilities are significantly more likely to report crime in their neighborhoods, live in moderately or severely inadequate housing compared to those without
Friedman 2023 (US) [53]	473,626 participants. (7,165 Medicaid beneficiaries with cognitive disabilities, all other people with disabilities with no Medicaid beneficiaries: n: 52,657) and nondisabled people (413,804) −7165 (65.1% female, 57% between 18 and 44 years old) disability type: cognitive Housing type: 66.6% renters, 33.4% own with a mortgage/loan	Secondary analysis of COVID-19 Household Pulse Survey by US Census Bureau data (housing insecurity, renting and owning, rent and mortgage payment) -no theory reported	−70% of Medicaid beneficiaries with cognitive disabilities were concerned about their ability to make their next housing payment -Increased sense of eviction among participants with cognitive disability -Among Medicaid beneficiaries with cognitive disabilities, renters and people with visual disabilities were significantly less confident about paying their next rent/mortgage payment on time
Fumarco 2017 (Italy) [19]	3 fictious household married couple tenants with visual disabilities, 1000 emails sent to housing ads a) without visual disabilities and no dog; (b) With visual disabilities and a guide dog; (c) Without visual disabilities and with a pet dog	Correspondence test field experiment (invitation to visit apartment, rejection) -No theory reported	-Tenants who have visual disabilities face higher rejection rates from advertisers than tenants without visual disabilities with no dog -Statistically significant gaps in invitations to visit the apartment between group A and B (visual disability and no dog vs visual disability with a guide dog) and group A and C (visual disability with no dog and no disability with a pet dog) -No statistically significant gap in invitation rates between groups B and C (visual disability with a guide dog vs no disability with a pet dog)
Garratt and Flaherty 2023 (UK) [54]	5 homeless adults with autism (60% female; age range 30–50+)	Visual qualitative methods (life mapping; life history interviewing) -No theory reported	-People with autism face ableist practices in homelessness experiences -Fewer opportunities to avoid homelessness: reduced family and friendship networks -Greater challenges to resolving homelessness: unmet needs in homeless hostels, limited access to social housing, barriers to navigating support services, risk of exploitation and crime
Grotti et al. 2024 (Ireland) [18]	**14,829 participants	Secondary analysis of Quarterly National Household Survey and 2014–2015 Survey on Income and Living Conditions (housing discrimination, housing deprivation, social groups)	-People with disabilities are significantly over- represented in local authority renting -People with disabilities report significantly higher levels of housing discrimination than those without -People with disabilities are at a significantly greater risk of housing deprivation than those without a disability
Hagner and Klein 2005 (US) [84]	148 mortgage underwriters	Randomized mailed applicant scenarios (earnings or public benefits) -No theory reported	-Applicants with a physical disability were significantly more likely to obtain a loan than an applicant with a developmental disability -Intensity of support and source of income did not affect probability of obtaining a mortgage
Hemingway 2010 (UK) [85]	**20 people with disabilities −10 representatives from disability organizations −60 representatives from housing and mortgage industry	Interviews (risk assessment of mortgage applications) -Theory: social model of disability	−17 respondents suggested some differences in the treatment of impairments that prevented people from securing a mortgage or making the process difficult -Severity of disability could affect what a mortgage broker was willing to lend were seen as higher risk -People with progressive impairments were viewed as having shorter life expectancy and potential loss of income that could affect loan risk
Heylen and Van den Broeck 2016 (Belgium) [86]	5 fictitious tenants in total, 684 online ads (phone) and 1769 online ads (control and one of the experimental tenants contacted each ad) -Tenant 1: Control; Tenant 2: Turkish or Moroccan name, Tenant 3: visual disability, Tenant 4: Single mother Tenant 5: Social assistance/ disability benefit; 100% male	Field behavioral experiment (discrimination, invitation to visit apartment) -No theory reported	-Discrimination/ selection was found in the email approach whereas people with disabilities were not found to be discriminated in the telephone approach
Heyman et al. 2023 (US) [55]	**464,732 people (23,871 or 5.1% with a disability) -Children: 49.34% male, mean age 6.67 years -Disability types: 68.35% emotional, 10.1% learning disability, 22.5% other medical conditions, 12.86% physical disability, 11.8% intellectual disability, 5.06% visual/hearing	Secondary analysis of 2018 national child abuse and neglect data system (housing as a caregiver risk factor) -Theory: social model of disability	-Parents with disabilities are significantly more likely to experience inadequate housing compared to parents without disabilities -The risk significantly increases for parents of children with intellectual disabilities as compared to parents of children with visual or hearing disabilities -Black parents with disabilities are significantly more likely than Black parents without disabilities to experience inadequate housing
Honey et al. 2017 (Australia) [56]	18 people with mental health conditions (55.5% female; mean age: 52 years) -Disability type: 100% mental health (depression, anxiety, post-traumatic stress, schizophrenia, bipolar disorder, schizoaffective disorder, undisclosed) -Housing type: 27.8% public housing, 22.2% community housing, 5.6% affordable housing, 22.2% private rental, 22.2% temporary housing	Interviews (experiences and perspectives about finding and maintaining housing, using housing assistance) -No theory reported	-Participants report difficulties applying for social services due to complex processes, long wait times, lack of transparency from workers and in policies, having to coordinate with multiple services, and short-time frames to make housing decisions -Landlords and housing services are not responsive to tenant repair requests, lack of communication on important matters -Housing insecurity and accommodation stress negatively impact tenants’ mental illness -Mental illness was an obstacle to acquiring resources to help with their housing needs
Kavanagh et al. 2016 (Australia) [57]	**1913 participants with a disability (56.2% female, majority above 30 years old) -Housing type: owner 49.3%, servicing a mortgage 27.8%, private renter 19.1%, public renter 3.9% and 13,037 observations in survey (no disability)	Secondary data analysis of national Australian surveys (mental health and wellbeing, housing and disability) -No theory reported	-Significant positive correlation between acquired disability as an adult and a decline in mental health and with unaffordable housing the decline is amplified -The largest negative contributor to mental health after disability acquisition is unaffordable housing
Korpela 1992 (Finland) [69]	204 children with cerebral palsy and at least one motor disability (54.9% female; mean age: 7.7 years) -Disability types: 60.3% cerebral palsy, 9.3% cerebral malformation, 5.9% myelomeningocele, 132% syndrome or chromosomal anomaly, 3.4% neuromuscular disease, 2.5% metabolic disease and 5.4% other diagnoses -Housing type: 66% renters, 34% unspecified	Interview and questionnaire (housing aids, modifications, care load) -No theory reported	−4% of participants lived in homes that have substandard equipment −17% in overcrowded homes, 66% in rental units. −54% of families living in block flats, had no access to a lift -Of the homes with lifts, 42% had problems of narrow doors, difficult use, and steps in front of lift -Toilets and washrooms need modification, families need more information on home modifications
May et al. 1991 (US) [58]	Two fictitious tenants reaching out to 100 landlords or rental agents of apartments -Tenant 1: Has visual disabilities -Tenant 2: Has mental health condition; 100% male	Phone survey (apartment availability) -no theory reported	−24% of landlords would not rent to a person with mental disabilities and 16% would not rent to a person with visual disabilities
Meschede et al. 2023 (US) [74]	**66,000 housing units (4956 who have a disability with needs, 7996 who reported having a disability without needs)	Secondary analysis of American housing survey (housing quality, neighborhood quality, housing affordability) -Theory: social model of disability	-Households with people with disabilities had the worst housing outcomes compared to households with people without disabilities -Households with people with disabilities are nearly twice as likely to miss their rental or mortgage payments and experience significantly higher severe housing cost burden than households with people without disabilities.
Mukoche et al. 2014 (Kenya) [59]	**50 people with physical and visual disabilities −9 people with disabilities, 4 landlords, and 2 researchers	Survey and focus group (housing conditions, access to housing) -No theory reported	-Lack of water borne sanitary facilities in the residential areas for people with disabilities -Roads and pathways were muddy and narrow to accommodate people with disabilities -Sewage and drainage systems were poor and posed risks to people with disabilities -Landlords lacked knowledge about disability
Nelson et al. 2013 (Australia) [60]	**27 mortgagors facing repossession (63% with a disability) and 9 service providers −87 surveys	Interviews, surveys (mortgage, financial distress, coping strategies) -No theory reported	-Interviewees reported a relationship between disability and financial distress, where disability can trigger financial distress and financial distress can trigger disability -People with disabilities reported that having a disability limited their options in coping with their mortgage defaulting
Nocon and Pleace 1997 (UK) [61]	**24 people with disabilities (58.3% female; age range: 20–50; 41.6% wheelchair users, 20.8% with mobility problems, 25% visually impaired, 4.2% with no vision 8.3% people with no hearing), 26 clinical and managerial staff	Survey and focus groups (housing needs) -No theory reported	-People with disabilities experienced issues related to security and vulnerability to crime -Many houses were too old/difficult to adapt -Lack of knowledge and negative attitudes towards people with disabilities from staff -Disagreements between people with disabilities and staff about type of work that needed to be carried out -Funding issues, finding appropriate properties
Nocon and Pleace 1998 (UK) [62]	**22 people with disabilities (between the ages of 18 and 64, 60% between 50–64 years) -Housing type: 40% owner occupiers, 35% local authority tenants, 4% housing associations, 2% privately renting, 1% residential homes, 10% with parents −26 clinical/housing staff	Interviews and survey (experiences with services, housing needs being met) -no theory reported	−75% of respondents reported having housing problems, with 29% of all respondents reporting they had one or more problems with housing condition (state of repair, cold, damp, or decoration) −39% of respondents needed housing adaptations and more appropriate housing -The input of people with disabilities is often not heard in the planning and provision of services
Nutsugbodo et al. 2022 (Ghana) [63]	173 landlords (55.5% male; 32% over the age of 60) **242 tenants (54.4% female; 78.1% under the age of 40) (disability status not reported)	Survey (discrimination test-based and statistical discrimination) -Social identity theory	-Disability was a significant determinant of discrimination against tenants by landlords
Omona 2023 (Uganda) [75]	**10,710 and 5,333 rural-urban households with and without disabilities, respectively from 12 subregions	Cross-sectional household survey (rurality and urbanism, housing standards, living conditions) -Theory: social model of disability, the human rights-based model and the citizenship theory	-Socio-economic status is higher among households without persons with disabilities −52% of rural households with people with disabilities report poles and mud for infrastructure, and more households without people with disabilities in urban areas have floors made of concrete -Lower level of living among households with members with disabilities was revealed both in urban and rural-based households
Plata 1979 (US) [87]	Three fictitious profiles reaching out to 92 advertisements for rental units −3 different groups:1) complex emotional needs 2) youth with a criminal history and 3) mental health and interviews with landlords about single mom with disabled child	Phone survey (apartment availability) -No theory reported	-Two negative responses were recorded to the youth with complex emotional needs -One negative response to the youth with a criminal history -One negative response to the youth with mental disabilities -Landlords reported fears of personal harm to others by the child with disabilities and property damage.
Quilgars et al. 2024 (UK) [64]	**35 people with learning disabilities who rent their own homes (85.7% social housing, 14.3% private housing) (age: 20–66)	Interviews (experiences finding and living in rental homes) -No theory reported	-Participants experienced a lack of available, accessible housing, lack of choice, lack of accessible information, high cost of accessible housing -Some had problems with heating, mold, noise, neighborhood behavior. -Repairs for accessibility was the biggest issue for most (issues with bureaucracy and delays) -Many enjoyed privacy, security and lack of surveillance, when renting privately
Sapey 1995 (UK) [65]	**31 people with disabilities (45% male; age range 18–60)	Survey, interviews (satisfaction with housing) -No theory reported	−45% had difficulty using all the rooms in their home -Difficulty obtaining funding for home modifications -Some people had difficulty getting the right advice
Saugeres 2011 (Australia) [66]	**20 people with disabilities 20 family members/caregivers	Interviews (housing decisions and priorities) -Theory: social model of disability	-Many private rentals are inaccessible and unaffordable -Accessible public housing has limited availability -Financial limitations: people with disabilities often excluded from the paid labor market -Often had to live further from services than desired -Lack of housing options
Schormans et al. 2024 (Canada) [70]	38 frontline staff working with youth and **11 homeless youth with intellectual disability	-Interviews, Participatory action project (forum theatre); day-to-day experiences, ability to secure housing supports) -Theory: conceptualization of vulnerability	-Youth with disabilities reported that homelessness is exacerbated by the disconnected service sectors (education, employment, medical systems), narrow requirements, lack of coordination and siloed services
Semeah et al. 2019a (US) [67]	39 veterans with various disabilities (82% male; age range 18–51+) -Housing type: 100% renters	Survey (process of renting, satisfaction with accessible features, awareness of fair housing act) -Bio-psychoecological model	-Lack of quality housing, quality of the neighborhood (lack of affordable housing in suitable neighborhoods), communication, policy and reintegration (challenge of finding housing after leaving military)
Semeah et al. 2019b (US) [68]	83 veterans with physical and/or psychological disabilities (82.9% male; ages 18–51+) -Disability type: 68% mental health condition, 44% report more than one disability -Housing type: 40% multi-family rental apartment or condominium, 20% single family home, 30% other (townhouse, mobile home, duplex, triplex)	Survey and interviews (discrimination, accessibility) -Theory of family housing adjustment	-Veterans with disabilities face challenges in locating and occupying suitable rental housing -Discrimination impacts housing satisfaction
Slavici 2023 (Germany) [88]	**50 semi-structured interviews (government and ministry officials, associations of the housing and building industry, people with disabilities, elderly and tenants, executives for building regulations, state subsidy banks and advice centers for accessible housing)	Mixed methods: Policy analysis, case studies, interviews (accessible housing) -No theory reported	-Political lobbyists have weak political influence in accessing decision-makers in housing politics -Accessible housing policy is rare -Advisory structures for accessible living are diverse and confusing
Soorenian 2013 (UK) [71]	**30 international university students with various disabilities and nationalities in (mean age 26) -Disability type: visual, physical, mobility, hearing, ADHD and dyslexia, mental health, myalgic encephalomyelitis -Housing types: 70% university-provided accommodation, 26.7% private housing, 3.3% live with partner	Focus group and interviews (intersectional identities, barriers to housing) -no theory reported	-Some experienced inaccessible amenities, put in undergraduate housing although being postgraduate leading to separation and isolation −13 participants unsatisfied with accommodations provided −6 reported having to move due to lack of accessibility and continuous need for negotiations for modifications. Some made their own modifications -Students with visual and physical disabilities are among the most dissatisfied with accommodations
Staples and Essex 2016 (UK) [89]	**34 parents of children with disabilities 8 developers/planners -Disability types (children): autism, cerebral palsy, developmental delay, epilepsy, Down syndrome, chromosome deletion, ADHD, William’s syndrome, trisomy, motor sensory neuropathy	Survey and focus group (current accommodation, housing design, barriers to inclusive housing) -No theory reported	-Deficiencies in mainstream housing increase stress for families with children with disabilities -Most housing was too small for needs of a family with a child with disabilities, needed adaptation, had poor access, lacked adequate storage space -Half the sample was unsatisfied with current accommodation and 75% wanted to move -Difficulties obtaining house accommodations -Institutional processes and negative attitudes preventing accessible housing design -Developers not always consulted about design requirement; housing needs were not assessed
Stone et al. 2023 (UK) [90]	10 autistic adults (80% male) Aged: 22–40, mean; 29.2 years -Disability Type: 100% autism Housing type: 100% hostels/shelters/temporary residences for homeless	Interviews (life history pathways through homelessness) -No theory reported	-Participants experience rough sleeping, couch surfing before accessing hostels -Experienced barriers to accessing services perpetuated homelessness (unsuitable accommodations and supports) -Some disengaged with services and preferred to sleep rough
Taylor 1989 (Canada) [91]	**66 people who have chronic mental disabilities living in hospitals, lodging homes and alone -Disability type: 64% schizophrenia, 11% manic-depressive, 8% schizo-affective, 5% personality disorder; Housing type: 28 lodging home, 17 independent living, 21 other	Interviews and questionnaire (living situation, length of residence, moving and reasons for moving) -No theory reported	−66 participants moved 236 times in two and a half years because of inadequate housing, overcrowding, uncleanliness, landlord problems -Higher level of education (high school) is correlated with higher desire to live independently, higher housing standards and less tolerance for marginalization -Clients who have chronic mental health condition are marginalized and ghettoized
Thomas 2004 (UK) [92]	**11 people with disabilities -Disability type: 100% mobility impairments, 8 full-time wheelchair users	Case study (experience of accessing the owner market) -No theory reported	-People with mobility impairments reported that they found it difficult to find a suitable property -People with mobility impairments encounter physical, attitudinal and systemic barriers -Real estate offices and websites are often inaccessible -Sales staff lack knowledge about disability and have negative and dismissive attitudes
Thurman et al. 2023 (US) [77]	18 disability or homeless service providers (61.1% female, mean age 44)	Interviews (perceptions and experiences) -No theory reported	-Participants struggled to provide appropriate, accessible services to people with intellectual and developmental disabilities (i.e., lack of training, awareness of needs, fragmented systems, inadequately funded health care and housing support)
Tomlin 2017 (US) [93]	1556 email inquiries sent to landlords with fictitious white, female profile (indicating disability or child)	Correspondence methodology (treatments: disability and single mother) -No theory reported	-People signalling disability receive 12.5% fewer responses about apartment rentals
Trippi et al. 1978 (US) [94]	**Two fictitious renters contacting 100 people advertising apartment for rent (landlords) -Renter 1: no details Renter 2: person with a disability who completed a training program ready for independent living	Correspondence methodology (treatment, apartment availability) -No theory reported	-Only 1% indicated a willingness to rent to a person with an intellectual disability −52% said the apartment was no longer available after finding out the renter had a disability −47% made the facility seem unattractive to rent −15% refused to rent to person with disabilities -The public was distrustful of training programs for independent living, asking questions about the potential tenants’ ability to take care of themselves, trustworthiness, temperament, and if they are harmful
Vaccaro 2019 (US) [78]	**31 people working in student affairs and have experience working with students with disabilities (61% female) in 21 higher education institutions −8 identified as people with disabilities	Focus groups (experiences working with people with disabilities, obstacles in campus housing) -Theory: ecological systems theory	-Challenges of balancing competing needs of students and community housing needs -Keeping personal privacy of students with disabilities when other students complain about preferential treatment -Meaning of reasonable accommodation is unclear -Problems with large dogs and unique service animals -Most campus housing staff lacked disability knowledge
Verhaeghe et al. 2016 (Belgium) [95]	Two fictitious renters contacting landlords and realtors of 268 properties on the Belgium rental housing market -Renter 1 (test): friend of a visually impaired person and a service dog; Renter 2 (control): no information about disability	Correspondence test (invited to visit a property or not) -No theory reported	-People with visual impairments are substantially discriminated against in the rental housing market -At least 1 in 3 discriminate against people with a visual impairment -Private landlords are at least twice as likely to discriminate against tenants with a visual disability than real estate agents -Realtors discriminate against one in five tenants with a visual impairment
White et al. 1994 (US) [76]	**16,305 households (17% households with a person with disabilities) 7,061 households (27.5% households with a person with disabilities)	Secondary analysis of two national studies/surveys (the Panel Study of Income Dynamics and the Survey of Income and Program Participation) (housing quality and housing affordability) -No theory reported	-Adults with a disability had less income, less employment and have higher percentage of being under poverty line -Households with a person with a disability are more likely to pay more than 35% or 50% for housing.

* Please note we only report key findings and outcomes related to the research question. We recognize that some of the terms used in the table are outdated and ableist; however, we retained the original terminology from the source articles to ensure accuracy.

**Article was missing demographic information related to age, disability type, sex, race, or housing type.

The studies used diverse methodologies including secondary data analyses [18,24,53,55,57,74,76,79,80,83], surveys [50,51,58,59,61–63,65,67–69,73,75,89,91], qualitative interviews [48,49,56,60,62,64–67,69–71,77,81,85,90,91], matched paired-test [23,52], correspondence methodology [19,84,86,93–95], audits of housing advertisements [82], focus groups [59,61,71,78,89], mixed methods [72,88], visual qualitative methods [54], case study [92], and participatory action research [70].

Study populations included people with disabilities [18,48,49,51,53–57,59,61,62,64–66,69,71,78,81,85,90–92,96,97], households [48,72,73,75,76] and housing units [24,74], family members or caregivers [66,89], landlords and housing providers [19,23,50,52,58,59,63,77,86,87,93–95], housing and mortgage industry representatives [60,84,85,88] planners and developers [89], clinical and housing staff [62,70,78] and disability organizations [85]. Twenty-eight studies reported the type of housing among participants and included people living in rental apartments or houses [19,50–53,58,61,63,64,68,69,86,93–95], homeowners or those attempting to purchase a house [53,57,60,62,68,81], people living with family [61], students in student housing [71,78] people in various housing types [51,56,57,62,82,91] and people experiencing homelessness [54,70,90].

Seventeen studies applied theoretical or conceptual frameworks including: ontological security theory [49], accessibility in eco-communities [72], person-environment framework [83], social model of disability [55,66,74,75,85], social identity theory [63], bio-psychoecological model [67], theory of family housing adjustment [68], conceptualization of vulnerability [70], ecological systems theory [78], independent living paradigm [51], spatial assimilation model [24], discrimination model [24], and human rights-based model and citizenship theory [75].

### Overview of findings

We organized our findings into three domains that reflect multi-processes consistent with socio-ecological theory: (1) ableism in finding and maintaining appropriate housing through renting and ownership; (2) factors affecting housing ableism; and (3) impacts of housing ableism (i.e., challenges with neighborhood environment) (see Table 2).

**Table:** 

Author, year (Country)	Ableism in finding and maintaining housing	Factors affecting housing ableism	Impacts of housing ableism
Anderson et al. 2024 (US) [79]	x		
Aplin et al. 2015 (Australia) [48]	x		
Aplin et al. 2020 (Australia) [49]			x
Aranda 2015 (US) [23]	x	x	
Beer et al. 2020 (Australia) [80]		x	
Bhakta and Pickerill 2016 (UK) [72]		x	
Burns 2004 (UK) [81]	x	x	
Callaway et al. 2021 (Australia) [82]	x		
Chakraborty et al. 2023 (US) [73]			x
Challe et al. 2023 (France) [50]	x	x	
Dawkins and Miller 2017 (US) [83]	x	x	
Dunn 1990 (Canada) [51]	x		
Flage and Le Gallo 2025 (France) [52]	x	x	
Friedman 2023 (US) [53]	x	x	
Friedman et al. 2018 (US) [24]	x	x	x
Fumarco 2017 (Italy) [19]	x	x	
Garratt and Flaherty 2023 (UK) [54]		x	
Grotti et al. 2024 (Ireland) [18]	x	x	
Hagner and Klein 2005 (US) [84]	x	x	
Hemingway 2010 (UK) [85]	x	x	
Heylen and Van den Broeck 2016 (Belgium) [86]	x		
Heyman et al. 2023 (US) [55]	x	x	
Honey et al. 2017 (Australia) [56]	x	x	
Kavanagh et al. 2016 (Australia) [57]	x		
Korpela 1992 (Finland) [69]	x		x
May et al. 1991 (US) [58]	x	x	
Meschede et al. 2023 (US) [74]	x		x
Mukoche et al. 2014 (Kenya) [59]	x	x	x
Nelson et al. 2013 (Australia) [60]	x	x	
Nocon and Pleace 1997 (UK) [61]	x	x	x
Nocon and Pleace 1998 (UK) [62]	x	x	x
Nutsugbodo et al. 2022 (Ghana) [63]	x		
Omona 2023 (Uganda) [75]	x	x	
Plata 1979 (US) [87]	x		
Quilgars et al. 2024 (UK) [64]	x		x
Sapey 1995 (UK) [65]	x		
Saugeres 2011 (Australia) [66]	x	x	x
Schormans et al. 2024 (Canada) [70]		x	
Semeah et al. 2019a (US) [67]	x	x	
Semeah et al. 2019b (US) [68]	x		
Slavici 2023 (Germany) [88]		x	
Soorenian 2013 (UK) [71]	x	x	
Staples and Essex 2016 (UK) [89]	x	x	
Stone et al. 2023 (UK) [90]	x	x	
Taylor 1989 (Canada) [91]	x	x	
Thomas 2004 (UK) [92]	x	x	
Thurman et al. 2023 (US) [77]		x	
Tomlin 2017 (US) [93]	x		
Trippi et al. 1978 (US) [94]	x	x	
Vaccaro 2019 (US) [78]	x	x	
Verhaeghe et al. 2016 (Belgium) [95]	x	x	
White et al. 1994 (US) [76]	x	x	

### Ableism in finding and maintaining appropriate housing

Eighty-four percent of studies reported ableism in accessing or maintaining housing including limited availability of suitable housing [18,24,48,51,55,59,61,62,64–69,71,74,75,78,79,82,83,89–92], barriers to accessing or viewing properties [19,23,50,52,56,58,63,64,66,81,82,84,86,87,90,92–95], and affordability challenges [53,60,64–67,74,76,83,84,90].

#### Limited availability of suitable housing.

Across twenty-four studies people with various types of disabilities faced challenges in accessing appropriate housing across rental and ownership contexts. Shortages of accessible units, limited opportunities for home modifications, and poor housing conditions were common [18,24,55,61,62,66,74,75,91]. Many participants lived in inadequate housing, including overcrowded, aging or unsafe dwellings [59,62,69,79,89,91]. Participants also reported challenges related to housing design [64,66–68,82,83,92]. Many homes lacked essential accessibility features and older housing stock often could not accommodate modifications [48,51,61,62,64,65,69,71,78,89]. Additionally, inaccessible layouts, narrow entrances and lack of elevators limited independence and usability [61,64,69,92].

#### Barriers to accessing or viewing properties.

Nineteen studies identified barriers during the housing search process, including limited information, physical inaccessibility, and discriminatory practices [19,23,50,52,56,58,63,64,66,81,82,84,86,87,90,92–95]. Participants often lacked clear and accessible information about available housing [23,64,82,92]. Housing advertisements frequently omitted accessibility details, and communication barriers limited access for some groups [64,82]. Meanwhile, physical barriers also restricted access to housing and real estate services [23,66,81,92]. Inaccessible offices, websites, and viewing processes reduced participation, particularly for individuals with mobility impairments [23,66,81,92].

Discriminatory attitudes among housing providers were also common [19,23,50,52,56,58,63,81,84,86,87,93–95]. Landlords, real estate agents, and builders often treated applicants with disabilities unequally [19,23,50,52,56,58,63,81,84,86,87,93–95]. Correspondence studies showed higher rejection rates for applicants with disabilities, including those with visual, mental, and intellectual disabilities [19,23,56,57,62,63,84,86,88,92,93]. In some cases, landlords were less likely to respond, refused to rent, or imposed additional requirements [94].

#### Affordability.

Housing affordability remains a major barrier for people with disabilities. Fourteen studies reported high housing costs, limited income, and difficulties accessing financial supports among people with disabilities [53,57,60,61,64–67,74,76,83–85,90]. Eight studies reported that people with disabilities experienced a high cost burden and were more likely to miss rent or mortgage payments [53,57,60,64,66,67,74,76]. Lower income and reduced employment opportunities further constrained housing options [53,57,76,83]. Six studies also identified barriers to obtaining loans and financial assistance [60,61,65,83–85]. Mortgage approval varied by disability type, with lower approval rates for individuals with developmental or cognitive disabilities [84,85]. Limited access to housing assistance programs further exacerbated affordability challenges [83].

### Factors affecting housing ableism

Thirty-six studies identified factors influencing housing ableism including limited provider knowledge [59,61,62,72,77,78,81,89,92], and intersectional influences such as socio-economic status [53,54,60,66,75,76,83], disability type [19,23,50,52,54,55,58,71,80,84,85,91,94], housing context [18,24,52,66,95], and systemic and policy barriers [54,56,67,70,72,77,88,89,92].

#### Limited knowledge of disability needs.

Nine studies showed that real estate agents, housing providers (e.g., landlords, builders, sales staff, housing service personnel) and clinicians lacked knowledge about accessibility requirements [59,61,62,72,77,78,81,89,92]. Builders, landlords, and service providers frequently misunderstood or overlooked the needs of people with disabilities [59,81]. This contributed to inadequate design, poor implementation of accessibility standards and negative or dismissive attitudes [61,77,89].

#### Intersectional factors affecting housing ableism.

Thirty studies reported findings on intersectional factors that affect housing ableism, including socio-economic status [53,54,60,66,75,76,83], type of disability [19,23,50,52,54,55,58,71,80,84,85,90,91,94,95], housing context [18,24,52,66,95], and systemic barriers [54,56,67,70,72,77,88,89,92]).

Seven studies showed that socio-economic status influenced housing access [53,54,60,66,75,76,83]. People with disabilities often had lower income, higher poverty rates and reduced access to housing assistance, increasing vulnerability to housing instability [54,60,66,76,83].

Fifteen studies reported that specific disability types were linked to unfair treatment in housing, particularly among renters [19,23,50,52,54,55,58,71,80,84,85,90,91,94,95]. People with visible disabilities [19,23,50,52,58,71,92,95], mental health conditions [52,58,80,85,91], or intellectual disabilities [55,80,84,85,94], or other less visible conditions [54,90] faced higher levels of discrimination.

Five studies reported how housing type influenced experiences of ableism [18,24,52,66,95]. Public housing often lacked accessible units, and private rentals were often inaccessible or unaffordable [24,66]. Some studies reported higher discrimination in private rental markets among real estate agents [52,95]. Meanwhile, nine studies reported how systemic and policy barrier further limited access [54,56,67,70,72,77,88,89,92]. For example, fragmented services, complex processes, and limited enforcement of accessibility policies created additional obstacles [56,72,89,92].

### Impact of housing ableism: Challenges with neighborhood environment

Ten studies examined the potential impacts of housing ableism and highlighted challenges related to the neighborhood environment [24,49,59,61,62,64,66,69,73,74]. People with disabilities often lived in areas with limited access to services, poor infrastructure and safety concerns [24,66]. Some studies reported higher exposure to crime, environmental hazards and natural disasters, as well as long distances to essential services [49,54,59,61,64,66,74]. These conditions often reduced quality of life and limited participation in daily activities [24,49,66,74].

## Discussion

Our review examined how people with disabilities experience housing ableism when seeking or maintaining independent housing. Addressing housing ableism is critical because housing is a basic human right and a key social determinant of health [6,10,57,98]. Previous reviews have focused primarily on institutional or supported housing settings [10,27–34], whereas this review provides a focused synthesis of independent housing contexts. This issue is especially relevant given ongoing housing supply and affordability crises across many countries [10].

Our interpretation of the findings is informed by the social model of disability and a socio-ecological perspective, which together emphasize that housing ableism arises from structural barriers and multi-level influences rather than individual impairments [44,45]. These models help to explain why barriers identified across studies, such as inaccessible housing, discriminatory practices and policies gaps, operate across interconnected systems, and reinforce inequities in access to independent housing.

We found that people with disabilities often lack suitable housing due to limited housing supply, poor housing conditions, and high costs. These conditions contribute to disproportionately high rates of homelessness and housing insecurity [99]. Limited accessible housing in both rental and ownership often forces people with disabilities to live in inadequate, or poorly located housing, frequently distant from essential services and public transit [100]. Inadequate housing conditions can increase risks of injury, social isolation, and unsafe living environments [10,22,27,38,39]. Inaccessible design, including limited space and missing accessibility features, further undermines safety and independence [101]. These conditions often reflect limited implementation of universal design and weak compliance with accessibility standards [101]. At the same time, structural economic factors, including lower wages and barriers to employment, further reduce the ability to secure appropriate housing [99,102,103].

Our review underscored that people with disabilities faced persistent barriers when accessing or viewing properties, including limited information, inaccessible environments and discriminatory practices. Housing listings and platforms often lack clear and standardized accessibility information, which complicates housing searches [82,92,104]. These findings highlight how housing systems often assume that independent living is designed primarily for people without disabilities. Discriminatory attitudes among landlords, property managers, and real estate agents further restricted access, particularly for individuals perceived as higher risk [52,85]. Limited knowledge of accessibility needs and perceived costs of accommodations often reinforced these attitudes [77,78].

Housing ableism varied across socio-economic status, disability type, housing context and policy environments. Lower income and reliance on social supports increased vulnerability and reliance on social supports increased vulnerability to housing instability [54,60]. People with visible disabilities, mental health conditions, or service animals often experienced discrimination early in the search and screening process, especially when requesting accessibility accommodations [23]. In contrast, people with invisible or less visible disabilities may initially avoid bias because their disability is not immediately apparent [105]. Housing context also influenced experiences. Rental housing and social housing often imposed barriers to modifications while private homeownership allowed greater flexibility but remained less accessible to many individuals [66]. Additionally, weak enforcement of accessibility policies further limited effective implementation, reducing policy impact [106]. When planners and builders overlook universal design, they often produce housing that restricts independence and reinforces exclusion [107].

Few studies within this review examined the direct impacts of housing ableism. However, some existing evidence indicates substantial consequences for neighborhood environments and quality of life [24,49,66,108]. Many people with disabilities lived in areas with limited services, poor infrastructure, and higher exposure to safety risks, including crime and environmental hazards [24,49,59,61,64]. These conditions arguably reduced opportunities for participation and social inclusion. Accessible and well-located housing can improve liveability by supporting social connection and independent participation in daily activities [108].

We also identified a gap in understanding the downstream consequences of housing ableism, including eviction. Although some studies suggest that people with disabilities experience increased financial stress and risk of housing instability [53], few studies explicitly examined eviction outcomes. Eviction can worsen health and wellbeing, increasing depression, anxiety and loss of access to services [109,110]. Additionally, policies such as nuisance ordinances may further disadvantage tenants with disabilities, forcing trade-offs between accessing care and maintaining housing stability [111]. These findings highlight that addressing housing ableism requires coordinated action across structural, policy, and environmental levels, consistent with the socio-ecological model, while reaffirming the social model of disability’s emphasis on removing systemic barriers to enable equitable access to independent housing.

### Implications

Our findings identify several implications for housing policy and practice. First, governments should increase the supply of accessible and affordable housing. Housing systems must recognize that people with disabilities are disproportionately affected by housing shortages and affordability challenges [3]. Policymakers should address gaps that limit housing supply and support broader adoption of universal design in new construction [10,112,113]. Governments should also provide targeted funding to support accessible housing development and home modifications. Second, policymakers and service providers should address the high rates of homelessness among people with disabilities. Expanding long-term supports, including supportive housing, can help individuals who are unhoused or at risk [103]. Addressing fragmented services and barriers to accessing supports is essential to improve housing stability.

Third, housing stakeholders should strengthen education, training and collaboration. Many housing providers lack knowledge of accessibility requirements and the experiences of people with disabilities. Increasing awareness among builders, landlords, real estate agents and lenders can improve compliance with accessibility standards and reduce discriminatory practices [114,115]. Fourth, housing practitioners should support collaboration and co-design in housing development [116]. Involving people with disabilities in planning, design, and decision-making can improve housing outcomes [30]. Expanding access to information about housing options and financial resources can improve access [117]. Centralized databases of accessible housing and tools that identify accessible housing may help match individuals with suitable units [118]. Housing listings and platforms should clearly report accessibility features, and housing services should ensure accessible communication and processes. Finally, policymakers and housing practitioners should prioritize inclusive approaches that reflect the needs and perspectives of people with disabilities [30]. Engaging individuals with lived experience throughout housing design and policy development can improve equity and long-term outcomes [30].

### Limitations, risk of bias and future directions

This review has several limitations. We did not register a review protocol, which may reduce transparency. Although we used a comprehensive search strategy, we may have missed some relevant studies, particularly from underrepresented regions such as Asia. We also excluded grey literature, so future studies should consider exploring policy and community level documents that capture housing-related ableism. Additionally, some researchers may view our inductive analytical approach as a limitation compared with a predefined deductive framework. Because our review covered many disability types, participant groups, methodologies, and housing contexts, countries, and decades, comparing findings was challenging and the results should be interpreted with caution. Given our review included diverse articles we could not always distinguish housing ableism from general housing barriers. Future studies should consider focusing on specific disability groups, particularly those with visible disabilities, who may face more distinct housing barriers. Our review covered many countries, each with different perceptions and treatments of people with disabilities, including supports, resources and policies, which can influence experiences of housing ableism.

Many studies in our review did not report on participants’ socio-demographic details, such as age, gender, race, type of disability, and type of housing, which limited our ability to understand how intersectional factors influence housing ableism. Future research should explore these factors in greater depth, especially since discrimination often intensifies for people with multiple minoritized identities [119]. Our review also highlighted a gap in understanding how housing ableism varies across age groups. More work is needed to the barriers and needs of youth, including supports for independent living. Additional research is needed to explore the different housing contexts, such as rentals, homeownership, homelessness, and how ableism manifests in each. Very few studies addressed rural housing and its impact on people with disabilities. We also found surprisingly little research on physical accessibility barriers when viewing or entering properties, an essential area for developing including housing solutions. Moreover, little is known about how discrimination affects the health and wellbeing of people with disabilities, highlighting an important area for future inquiry. Policymakers and researchers should prioritize strategies to mitigate housing ableism and develop standardized measures, capturing both direct and indirect forms of ableism at individual and structural levels to strengthen future evidence.

## Conclusions

Our review synthesized the literature on disability-related discrimination (ableism) among people seeking or maintaining independent housing. Our findings identified three main categories. First, people with disabilities routinely encounter ableism when seeking or maintaining housing, including a lack of suitable options, barriers to accessing or viewing properties and affordability challenges. Second, several factors contribute to housing ableism, such as limited knowledge about disability and intersectional influences including socioeconomic status, disability type, housing type and systemic barriers. Third, housing ableism can negatively affect people’s lives, particularly through neighborhood-level challenges. These findings highlight the urgent need to address ableism across the housing sector. Future research should focus on developing solutions and interventions that reduce the multiple forms of housing ableism experienced by people with disabilities.

## Supporting information

S1 Fig Sample search strategy. (DOCX)

S2 Fig PRISMA-ScR Checklist. (DOCX)
