# Supplementary material for: Housing ableism in finding and maintaining housing among people with disabilities: A scoping review
Source: PLoS One. 2026 Jun 10;21(6):e0351309. doi: 10.1371/journal.pone.0351309 (PMC13252812; doi:10.1371/journal.pone.0351309)
Supplement: S1 Fig — (DOCX) [file pone.0351309.s001.docx]

**S1. Search strategy**

**Database: Embase Classic+Embase <1947 to 2025 May 22>, APA PsycInfo <1806 to May 2025 Week 3>, Ovid Healthstar <1966 to March 2025>, Ovid MEDLINE(R) ALL <1946 to May 22, 2025>**
**Search Strategy:**
**1**  house.mp. (287264)
**2**  supported living.mp. (696)
**3**  exp Independent Living/ or dwelling.mp. (195651)
**4**  apartment.mp. or exp Housing/ (122576)
**5**  condominium.mp. (284)
**6**  residential building.mp. (920)
**7**  home.mp. (1335851)
**8**  living environment.mp. (12185)
**9**  independent living.mp. or exp Independent Living/ (65994)
**10**  inclusive housing.mp. (44)
**11**  housing standards.mp. (251)
**12**  home environment.mp. or exp Home Environment/ (47761)
**13**  1 or 2 or 3 or 4 or 5 or 6 or 7 or 8 or 9 or 10 or 11 (1881959)
**14**  ableism.mp. or exp Disability Discrimination/ (19849)
**15**  discrimination.mp. or exp Discrimination Learning/ or exp Disability Discrimination/ or exp Social Discrimination/ or exp Discrimination, Psychological/ (766100)
**16**  exp Bias/ or exp Prejudice/ or social bias.mp. (299505)
**17**  social stigma.mp. or exp Social Stigma/ (83375)
**18**  social exclusion.mp. or exp Social Isolation/ (113426)
**19**  bullying.mp. or exp Bullying/ (58230)
**20**  harassment.mp. (30940)
**21**  oppression.mp. (18946)
**22**  exp Aggression/ or microagression.mp. (235551)
**23**  marginalization.mp. or exp Social Marginalization/ (23413)
**24**  14 or 15 or 16 or 17 or 18 or 19 or 20 or 21 or 22 or 23 (1488543)
**25**  disabled persons.mp. or exp Persons with Disabilities/ (248846)
**26**  exp Intellectual Disability/ or disability.mp. (1903092)
**27**  disab*.mp. (1605726)
**28**  functional limitation.mp. (10920)
**29**  physical limitation.mp. (2379)
**30**  sensory impairment.mp. (7830)
**31**  motor disorder.mp. or exp Motor Disorders/ or exp Spinal Cord Injuries/ (1368064)
**32**  hearing impairment.mp. or exp Hearing Loss/ (339132)
**33**  exp Persons with Visual Disabilities/ or exp Vision Disorders/ or vision impairment.mp. or exp Vision, Low/ (503147)
**34**  wheelchair user.mp. (1634)
**35**  exp Autism Spectrum Disorder/ or developmental disorder.mp. or exp Autistic Disorder/ (248059)
**36**  brain injury.mp. or exp Brain Injuries/ (533223)
**37**  motor disorder.mp. or exp Motor Disorders/ or exp Spinal Cord Injuries/ (1368064)
**38**  neurodiverse.mp. (793)
**39**  exp Orthopedic Procedures/ or orthopaedic condition.mp. or exp Musculoskeletal Diseases/ (6077787)
**40**  amputation.mp. or exp Amputation, Surgical/ (199298)
**41**  cerebrovascular disorder.mp. or exp Cerebrovascular Disorders/ (1877546)
**42**  handicap.mp. (50709)
**43**  head injury.mp. or exp Craniocerebral Trauma/ (767299)
**44**  impairment.mp. (1652773)
**45**  exp Amputees/ or limb loss.mp. or exp Artificial Limbs/ (46892)
**46**  25 or 26 or 27 or 28 or 29 or 30 or 31 or 32 or 33 or 34 or 35 or 36 or 37 or 38 or 39 or 40 or 41 or 42 or 43 or 44 or 45 (12796695)
**47**  13 and 24 and 46 (9007)
**48**  limit 47 to human [Limit not valid in Ovid MEDLINE(R); records were retained] (8604)
